# Children’s Physical Activity during COVID-19 in Ontario, Canada: Parents’ Perspectives

**DOI:** 10.3390/ijerph192215061

**Published:** 2022-11-16

**Authors:** Monika Szpunar, Kendall Saravanamuttoo, Leigh M. Vanderloo, Brianne A. Bruijns, Stephanie Truelove, Shauna M. Burke, Jason Gilliland, Jennifer D. Irwin, Patricia Tucker

**Affiliations:** 1 Health and Rehabilitation Sciences Program, Faculty of Health Sciences, University of Western Ontario, London, ON N6A 5B9, Canada; 2 School of Occupational Therapy, Faculty of Health Sciences, Elborn College, University of Western Ontario, 1201 Western Road, London, ON N6G 1H1, Canada; 3 ParticipACTION, 77 Bloor Street West, Suite 1205, Toronto, ON M5S 1M2, Canada; 4 Member Interest Groups Section, Professional Development and Practice Support, College of Family Physicians of Canada, Mississauga, ON L4W 5A4, Canada; 5 School of Health Studies, Faculty of Health Sciences, University of Western Ontario, London, ON N6G 1H1, Canada; 6 Children’s Health Research Institute, Lawson Health Research Institute, 750 Base Line Rd E, London, ON N6C 2R5, Canada; 7 Department of Geography and Environment, University of Western Ontario, London, ON N6G 1H1, Canada; 8 Department of Pediatrics, University of Western Ontario, London, ON N6G 1H1, Canada; 9 Department of Epidemiology & Biostatistics, University of Western Ontario, London, ON N6G 1H1, Canada

**Keywords:** children, COVID-19, Ontario, parents, sport, play

## Abstract

The COVID-19 pandemic has had a large influence on children’s physical activity (i.e., play and sport) opportunities. The purpose of this study was to describe parents’ perspectives of their children’s (ages 0–12) physical activity experiences during the pandemic (i.e., since the onset in March 2020 until follow-up survey completion date [between August to December 2021]). As part of the ‘Return to Play’ study conducted in Ontario, Canada, two-parent report surveys were completed online via Qualtrics. Surveys measured parents’ perspectives regarding their children’s physical activity since the onset of the pandemic (*n* = 17 items) and collected demographic information (*n* = 16 items). Open-ended questions were included to gather a rich understanding of parents’ experiences (i.e., supports, challenges) with facilitating their children’s physical activity. Descriptive statistics were calculated to describe parents’ perspectives of their children’s physical activity experiences and to determine parent demographics. Open-ended questions were analyzed via deductive content analysis. Parents (*n* = 382) reported that they noticed behavior changes in their children because of the pandemic (65.9%), and most (73.7%) reported challenges with supporting their children’s activity during periods when public health measures were in place. Many parents (44.5%) stated that their children asked about returning to play/sport more than three times per week during periods when play/sport facilities were closed in Ontario. Qualitative data identified common supports parents used (e.g., getting active outdoors, forming mini social ‘bubbles’), and challenges they faced (e.g., work, children’s increased screen time, and home schooling), pertaining to their children’s physical activity.

## 1. Introduction

On 11 March 2020, coronavirus disease (COVID-19), caused by the SARS-CoV-2 virus, was declared a global pandemic by the World Health Organization (WHO) [1]. As a result of the severity and transmissibility of the disease, many countries imposed public health protections to protect the safety of communities. In Canada, some of these protections included mask mandates, hand sanitization protocols, and physical distancing requirements [2]. Specifically in Ontario, where COVID-19 case counts were among the highest in the country, and some of the most stringent public health protections in the country were put in place [3], studies exploring children’s movement revealed large declines in physical activity, including outdoor play and time spent outdoors [3,4,5,6]. 

Prior to COVID-19, approximately 78% of children (ages 5–19) in Canada were enrolled in some form of organized sport and/or physical activity-related activity [7], while only 21% of children (ages 5–11) were engaging in unstructured forms of play [8]. However, since the onset of the COVID-19 pandemic, the extended closures of various facilities that support children’s sport engagement (e.g., sport facilities, community centres), and school programs such as intramural sports, researchers have identified a shift toward children’s increased engagement in unstructured activities (e.g., playing outdoors, playing at home) [9,10,11]. For example, Pelletier and colleagues [11] found that during a period of early COVID-19 (September to December 2020) parents in Canada and their children participated in increased unscheduled and imaginative play, including more time spent playing outside in the yard and/or in unsupervised areas of the home (e.g., basement, garage). Similar findings (e.g., increase in unstructured physical activity) have been observed in other countries (i.e., United States; [9]). Clearly, the pandemic has had an impact on physical activity (i.e., play and sport) experiences for children and families, requiring families to become more creative regarding their scheduling of movement opportunities. 

As COVID-19 case counts have fluctuated across Ontario over the last two years [12], some sport/extracurricular activities have been able to re-open with enhanced safety protections, especially during periods when case counts were lower in the province [13]. More specifically, since March 2020, the Ontario government has consistently published updated rules and regulations regarding the types of settings permitted to be open to the public, and these public health regulations have largely varied based on city and risk of transmission [12,13]. During the first year of the pandemic, government officials closely monitored case counts and put cities into 1 of 3 phases of re-opening (Ontario’s re-opening framework during this study included 3 phases: Phase 1—protect and support; Phase 2—restart; and Phase 3—recovery. Cities were assigned a phase based on the number of positive COVID-19 cases present in the community. Sports and/or community centers were only permitted to open in phase 2.) [13], and these phases dictated the types of activities and settings that were open to civilians (e.g., sports arenas, shopping malls, outdoor playgrounds). Some cities with larger population densities (e.g., Toronto) faced longer periods of closures, with closures occurring in the winter months (i.e., November–February 2020–2021) being in place for the longest periods. However, during the warmer months of the COVID-19 pandemic in Ontario (i.e., May to September 2020–2021), when individuals were more likely to engage in outdoor sports, host gatherings outdoors, and case counts tended to be lower [14], many facilities were permitted to re-open (e.g., outdoor sport facilities and community centers) [14]. During these periods of reduced risk of transmission, and lower COVID-19 case counts in 2020 and 2021, Ontario’s government encouraged families to gather outdoors, and form mini bubbles (i.e., small social circles with the same people over time) of 10 people or less, in which members did not have to physically distance [14]. These bubbles were recommended to support citizens’ physical and mental health, and to reduce feelings of social isolation [14]. The many changes to public health protections in Ontario over the course of the pandemic have resulted in some children returning to their play and sport activities. For children in their younger years who are unable to make choices on their own, these decisions were largely driven by their parents.

Given that parents act as gatekeepers to children’s unstructured (e.g., outdoor play) and structured physical activity opportunities such as organized sports (e.g., responsible for registering, financing, and providing transport) [7,15], it is probable that parents’ opinions and/or beliefs regarding the pandemic will influence their children’s opportunities to return to play/sport throughout the pandemic. Therefore, it is important that parents’ perspectives regarding their children’s activity during varied periods of public health protections and virus outbreak (e.g., higher, and lower-case counts, emergence of variants) [12,16] are well understood. The purpose of this study was to describe Ontario parents’ perspectives regarding their children’s physical activity experiences from the start of the pandemic (March 2020) until their follow-up survey completion date (between August to December 2021). Specifically, parents were asked to reflect on whether they observed any changes in their children’s play/sport engagement over the course of the pandemic, noticed any behavioral shifts, their perspective on virtual sport opportunities, and to report on their level of concern about their children’s physical activity levels during (The statement during the pandemic used throughout this paper refers to the period between the declaration of the COVID-19 global pandemic in March 2020 up to the participants’ follow-up survey completion date (i.e., between August–December 2021)) and following the pandemic. A secondary objective was to gather insight regarding parents’ experiences (i.e., supports, challenges) with supporting their children’s activity.

## 2. Materials and Methods

### 2.1. Study Design and Procedures

**Figure 1:**
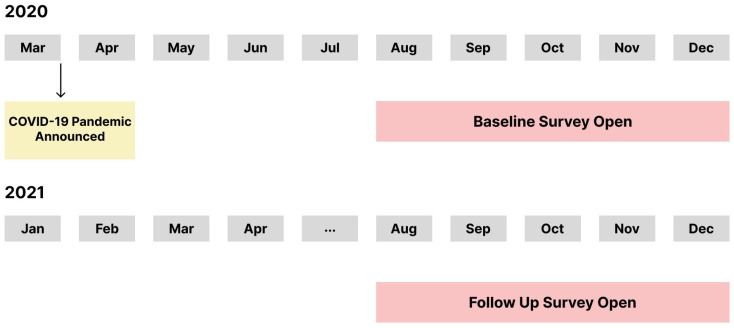
Timeline of the Return to Play Study surveys.

The current paper is a part of the larger ‘*Return to Play*’ study (details published elsewhere) [10,17], and presents a cross-sectional analysis of the larger repeated measures study. This study employed two online Qualtrics surveys (i.e., baseline survey; August to December 2020 and follow up survey; August to December 2021) to assess parents’ perspectives of their children’s return to play/sport during the COVID-19 pandemic. See Figure 1 for a study timeline, and Supplementary File S1 for items from the *Return to Play* surveys used for this paper. *Return to Play* was defined for the purpose of this study as encompassing both unstructured (i.e., playing in the neighbourhood) and structured (i.e., organized sport) activity post-COVID-19 pandemic. Ethics approval was provided by the Non-Medical Research Ethics Board at the University of Western Ontario (REB #116331).

### 2.2. Recruitment and Participants

Social media platforms (e.g., Twitter, Facebook) were used to recruit parents across Ontario. Sport organizations were also contacted and invited to share study details with their respective communities. Participants were eligible to participate if they were a parent and/or legal guardian living in Ontario with a child (or children) aged 12 and under; had custody of their children at least 50% of the time (at the time of the baseline survey); and were fluent in English. Parents with children aged 12 years and under were included to capture perspectives of parents with young children (e.g., 0–5 years) and school-aged children (e.g., 6–12 years).

### 2.3. Instruments and Tools

Questionnaire items (for the larger *Return to Play* study) were created by the research team and were informed by the COVID-19 situation in Ontario, Canada at the time of survey creation [17]. Online tools, administered at baseline (August to December 2020; *n* = 162 survey items) and follow-up (August to December 2021; *n* = 58 survey items), were completed by parents. The baseline survey collected demographic information and assessed Ontario parents’ perspectives on their children’s (≤12 years) physical activity-related behaviors prior to and during the pandemic to date (e.g., amount of time children spent engaged in physical activity prior to the COVID-19 pandemic), as well as their return to play/sport plans and attitudes for their children during various time points throughout the pandemic (e.g., sports/play activities they intend to return to once permitted) [10,18]. The follow-up survey employed similar items to assess how parents’ perspectives have changed over time (e.g., sports/play activities they have returned their children to) with the addition of new items to capture how children’s movement has (or has not) changed since the onset of COVID-19. This paper presents demographic information of participants collected at baseline, as well as results from a subset of the second survey (*n* = 17 items) that asked parents to reflect on their children’s physical activity experiences since the start of the pandemic (March 2020) up until their follow-up survey completion date (August to December 2021). Completion of the online survey indicated consent to participate.

#### 2.3.1. Demographic Questionnaire

The demographic questionnaire in the *Return to Play* study included sixteen items, capturing information such as parents’ and children’s age, gender, ethnicity, and household income. 

#### 2.3.2. Parents’ Perspectives of Their Children’s Physical Activity Experiences and Behaviors 

Seventeen questions assessing parents’ perspectives om their children’s physical activity experiences (i.e., play and sport) and behaviors during COVID-19 were measured via the follow-up survey. Questions were asked using a variety of formats, including multiple choice (i.e., select all that apply), open-ended items, and yes/no/prefer not to answer. Specifically, parents were asked how often their children asked about returning to their pre-COVID activities (e.g., play/sport), and whether their children were interested in the same activities they engaged in pre-COVID. Further, parents were asked to identify their level of engagement in physical activity alongside their children during the pandemic to date, and the supports/challenges they experienced while promoting their children’s physical activity. Parents were also asked whether they noticed any differences in their children’s physical activity levels on weekdays versus weekends, and behavior changes since the onset of the pandemic. Finally, parents were asked about their perspectives on virtual sport opportunities and were asked to report on their level of concern regarding their children’s physical activity levels following the COVID-19 pandemic. Open-ended questions were embedded as a part of the follow-up survey to allow parents to elaborate on details, share in-depth opinions, and express their thoughts regarding their observations of their children’s physical activity experiences.

### 2.4. Data Preparation and Analysis

To describe the participant demographics and parents’ perspectives regarding their children’s physical activity, descriptive statistics, including means and standard deviations, were computed in SPSS (version 27) [19]. Survey responses with more than 15% missingness (i.e., incomplete data) were removed [20]. Participant responses to open-ended questions were uploaded to QSR NVivo (version 12) [21] and were independently and deductively coded [22] by two researchers to ensure confirmability and to identify common responses [23]. 

## 3. Results

### 3.1. Demographics

Of the 382 parents with complete data for the Return to Play survey at time 2 (i.e., survey 2), demographic information was available for 223 participants. Most participants identified as female (94.6%), Caucasian (88.3%), lived in a detached house (79.4%) and worked full-time (71.3%). The average age of participants’ children was 6.7 years (*SD* = 3.2). See Table 1 for full participant demographics.

### 3.2. Parents’ Perspectives of Their Children’s Physical Activity Experiences and Behaviors 

When prompted to reflect on the impact of the COVID-19 pandemic on their children’s physical activity, just under half of parents (44.5%) reported that their children asked about returning to sport more than three times per week during periods when sports/play were deemed inaccessible in Ontario. Many children were either interested in re-joining the same sports (40.4%) or interested in the same sports that they had engaged in prior to the pandemic with the addition of new ones (47.0%). During times when supportive spaces (e.g., parks, sport facilities) were closed, 39.8% of parents reported engaging in activity often (i.e., weekly) with their children, while 31.2% of parents reported engaging daily. Nearly all parents (80.1%) identified that having access to outdoor spaces helped them to support their children’s activity during these times, and 71.7% reported forming mini bubbles; however, most parents (73.7%) also reported experiencing challenges with supporting their children’s activity during periods of strict public health protections (e.g., stay at home periods) in Ontario. Most parents (75.1%) reported that they were not interested in continuing virtual sports/activities, and many (65.9%) reported that they noticed behavior changes in their children because of the pandemic (described further by participants via open-ended items, seen below). Lastly, regarding parents’ level of concern about their children’s physical activity post-pandemic, 22.9% of parents reported feeling very concerned, while 36.3% of parents reported feeling somewhat concerned. See Table 2 for complete details regarding parents’ perspectives of their children’s physical activity experiences.

#### Parents’ Responses to Open-Ended Items 

Open-ended responses revealed common challenges and supports identified by parents in association with promoting children’s physical activity. Frequently noted challenges included a lack of space and/or equipment inside or outside participants’ homes, children missing their peers, lack of time and work demands, and parental burnout/inability to co-participate in activity with their children. In contrast, supports identified by parents included access to outdoor space, support from family and friends, their personal interest in remaining active and supporting their children’s activity (i.e., motivation), and access to sport- and play-related resources. Common responses from parents regarding the types of behavior changes they noticed among their children included a lack of socializing (e.g., with peers and/or family members), anxiety towards social situations, heightened agitation, increased general sadness/sense of loss, increased screen time, and parental separation anxiety. Further, responses to open-ended questions also revealed why some parents were not interested in virtual opportunities, citing increased screen time and the lack of perceived social, physical, or developmental benefits from these opportunities as common reasons. Some parents also reported that their children did not enjoy virtual play/sport activities. However, select parents did enjoy virtual programming for their children, referencing that it was a safer option and more convenient (e.g., in poor weather, saves time). When asked about changes to their children’s physical activity levels following their return to sport/play activities, some parents noted that their children had not yet returned (e.g., of because waiting for vaccination (COVID-19 vaccines for children aged 5–11 and children aged 6 months to 5 years were approved on 19 November 2021 and 14 July 2022, respectively. Please see the following website for a specific breakdown of timelines and vaccine approval in Canada: https://www.cihi.ca/en/canadian-COVID-19-intervention-timeline, accessed on 5 November 2022)) or that they did not notice any changes if they had returned. Alternatively, some parents reported noticing positive changes in their children’s physical activity levels since returning (e.g., more active, more motivation to get active, better sleep), while others described decreased strength and endurance when compared to pre-COVID. Most parents reported that their children engaged in increased activity during the weekends, as weekdays were flooded with work from home demands for parents, and online schooling for children. See Table 3 for all questions, common responses and supporting quotes from parents relating to their children’s healthy movement behaviors.

## 4. Discussion

The purpose of this study was to describe parents’ perspectives regarding their children’s physical activity experiences during a period of the COVID-19 pandemic (i.e., March 2020 to time of follow-up survey completion; between August–December 2021) in Ontario, Canada. Parents reported taking time out of their schedules on a weekly basis to facilitate their children’s activity during periods of strict public health protections (e.g., stay at home periods), mixed feelings about virtual physical activity opportunities, and that the pandemic led to behavioral changes in their children. Factors that helped to support children’s activity during the pandemic (e.g., access to outdoor space), and challenges experienced (e.g., lack of time and work demands), were also explored. The results of this study highlight important considerations regarding support and resources needed for parents with children during the pandemic period. Several findings are discussed below.

Most parents reported that having access to outdoor spaces helped them to support their children’s activity during periods of strict public health protections (e.g., stay-at-home periods) in Ontario. This finding is consistent with other research conducted over the course of the pandemic in Canada, which identified outdoor spaces as being a facilitator for promoting children’s activity [4,5,10,11]. In addition to outdoor space being identified as a facilitator, parents also addressed the role of support from family and friends to encourage physical activity among their children. This is meaningful, as research has highlighted the negative impacts of increased social isolation among children during the pandemic [24], reiterating the importance of maintaining social relationships during such times. Furthermore, nearly three-quarters of participants reported forming mini social ‘bubbles’ with other families and/or children during periods of strict public health protections. Given the evidence surrounding the importance of social relationships towards mental and physical health [25,26] and the lack of connection experienced by many during the pandemic, it is likely that families gravitated towards these mini-bubbles as means of reducing feelings of social isolation. Although this study did not capture the nature of gatherings among those who formed mini-bubbles (e.g., how often and how), these social interactions might have also acted as facilitators to increase children’s physical activity levels, as research shows that children are more active when they are with their peers [27,28].

Many parents listed access to resources and equipment as a support to their children’s physical activity, which brings about important considerations regarding income and/or job stability, particularly as some parents have reported increased financial barriers during COVID-19 [10]. Further, studies exploring the influence of housing type and neighborhood have revealed that having limited space (e.g., small backyard, apartment living) can act as a challenge to supporting children in physical activity [10,29]. These findings suggest that, dependent on context and the built environment, space and equipment can either serve as supports (i.e., if had access to space) or barriers (i.e., if had limited access). This will be an important future consideration as it is clear that the COVID-19 pandemic has impacted individuals disproportionally [30]. Finally, many parents reported that their own personal interest in physical activity helped them to engage in movement with their children. This finding is consistent with previous research, that has found parents who were physically active on their own schedules tended to have more active children [31].

With respect to virtual physical activity opportunities, many participants in the present study reported that they were no longer interested in continuing these activities. This is contradictory to qualitative findings from our *Return to Play* study, where child and parent participants reported being interested in virtual sport/play [10] In addition, other research conducted shortly after the onset of the pandemic also showed that many parents reported relying on virtual opportunities to support their children’s physical activity [4,9,10]. It is likely that some parents, at the time of completing this survey (more than a year into the pandemic), may have started to experience fatigue regarding virtual opportunities. For example, children in Ontario were required to engage in online learning (e.g., away from school) for many months during the COVID pandemic (in a staggered format, when in-person learning during COVID-19 was deemed unsafe by the provincial government), prior to the time this study was launched [32]. As such, the novelty of virtual physical activity and/or extracurricular opportunities may have worn off due to other virtual demands, such as children’s daily online learning activities on the computer. This is supported by a study conducted in China that assessed parents’ (*n* = 1062) and children’s (*n* = 738) perspectives on virtual opportunities, and identified that the majority of parents (57.4%) and children (62.3%) reported feeling inadequately supported in the virtual learning environment (e.g., not enough teacher to student interaction), and that screen-time had notably increased with online schooling (e.g., exceeding 3 h daily), likely contributing to parental fatigue of online platforms [33]. Open-ended items from this study also showed that parents felt that their children needed time away from screens, given the large portion of time spent online and a preference for in-person socialization; however, some parents still reported using virtual opportunities to support their children’s movement and referenced time saved and safety as reasons for doing so. The lack of social interaction in the online environment and high screen time among children could account for why many parents did not want to return to online platforms for physical activity opportunities.

Many parents revealed that they struggled to balance their own work-from-home demands while additionally managing their children’s online schooling (e.g., assisting children to set up computers, ensuring they stayed focused), resulting in their children engaging in less physical activity throughout the week (compared to the weekend) due to a lack of time. Previous COVID-19 pandemic research has shown that parents, particularly mothers, have taken on additional roles during weekdays (e.g., supervising their child while in online schooling) [34]. The large shift in daily routines for families undoubtedly affected children, and likely parents of children, as parents reported notable changes to their children’s behaviors. More specifically, similar to other research that transpired during the pandemic [4,5,35], parents reported increased screen time and desire to remain on screens among their children. With reference to mood, parents found that their children were more easily agitated by situations, were more easily saddened, and described a sense of loss. Furthermore, parents in this study noted that children appeared to exhibit increased anxiety toward social interactions and, in some cases, experienced separation anxiety (or clinginess). This finding is supported by a rapid review indicating that during COVID-19, children have been more irritable, clingy and experience increased anxiety [36]. Clearly, there is a need to support parents and offer strategies for reducing these types of feelings among their children.

Other barriers that parents faced included their own burnout and/or inability to engage in activity with their children. For instance, parents reported disability, poor mental health, exhaustion, and/or poor physical fitness as reasons for not being able to participate in activity with their children. Given that poor mental health and heighted anxiety presented among parents during the pandemic [37], this is a concerning finding. Research has also shown co-participation to be an important contributor to children’s activity during and prior to the pandemic [4,31], and studies have shown that children are more likely to drop out of sport when they have inactive parents [38]. Furthermore, as changes to children’s physical activity are unlikely without parental support [39], there is a need for additional support for parents to encourage co-participation during this time.

More than half of participants reported feeling *very concerned* or *somewhat concerned* regarding their children’s physical activity levels following COVID-19. Studies such as those conducted by Moore et al. [4,5] and Kharel et al. [40] have revealed drastic declines in Canadian children’s physical activity over the course of the pandemic. For example, Moore et al. identified that 18.2% of children (*n* = 1472) were meeting guidelines in April 2020; [4] however, at 6-month follow up (i.e., October 2020) [5], this number dropped to 14.3% among the same cohort of children. Furthermore, given the long amount of time away from play and sport activities for many children who were not keen or able to engage at home, there may be disproportionate effects on fitness levels. As the pandemic continues, increased supports are needed for parents to ensure their children are engaging in adequate activity. Future research should focus on identifying sustainable physical activity supports that are enjoyed by both parents and children beyond those that are offered virtually. In addition, physical activity supports that are feasible (i.e., affordable, transportable) should be identified to provide relief for parents of young children in the case of extended stay-at-home periods.

Strengths of the present study include the repeated measures design and the inclusion of both qualitative and quantitative data. In terms of limitations, the sample was drawn from the larger Return to Play study, and more than half of the participants were lost to follow-up, resulting in a smaller sample size than intended. Second, demographic data were not available for all participants, since 42% of participants inserted the wrong unique identifier codes when completing the second survey, rendering it impossible for researchers to match participant responses across time points. Furthermore, participants in this study were primarily Caucasian women from double-parent households with higher-than-average household incomes, representing a highly homogenous and affluent population, which may skew the perspectives and reduce generalizability of the findings.

## 5. Conclusions

Consistent with other research, [4,5,10,11] access to the outdoor environment and ability to engage in activity outdoors (e.g., in parks, outdoor playgrounds, backyards at home) are strong facilitators in supporting children’s physical activity during a pandemic. Further, behavioral changes in children, such as increased screen time and social anxiety during the COVID-19 pandemic have been observed by parents, resulting in ongoing concerns about their children’s physical activity as the pandemic continues in Ontario. Perspectives with regard to virtual opportunities to support their children’s physical activity were also reported as non-favorable, due to their association with increased time spent on screens and the lack of social connectedness offered/possible. As public health protections continue to change in Ontario, alongside the prevalence of the COVID-19 virus, future investigations are needed to identify what types of supports can be put in place for parents to ensure they can adequately support their children’s physical activity in ways that are safe, accessible, affordable, and engaging. For example, public health professionals should investigate offering financial support for parents to purchase new equipment (e.g., bikes, trampolines) that can support their children’s physical activity while at home. Financial support could be offered at a family specific (e.g., number of children per household) and age-specific level (e.g., dependent on the age of children). In addition, if schools were to return to remote learning, increased efforts should be placed on physical education for children. The role of small social bubbles was clear within our findings; therefore, future research should consider the risk–benefit these social relationships offered during times of viral transmission. In addition, mini bubbles should be explored further regarding their role in supporting families during periods of extended social isolation.

## Figures and Tables

**Table 1:** Participant Demographics *(n =* 223).

	*M*	*SD*
Parent Age (years)	39.0	5.6
Child Age (years)	6.7	3.2
	***n***	**%**
Parent Gender		
Male	11	4.9
Female	211	94.6
Transgender	1	0.4
Children’s Biological Sex		
Male	73	32.7
Female	150	67.3
Type of Living		
Rural	50	22.4
Suburban	101	45.3
Urban	72	32.3
Ethnicity		
Caucasian	197	88.3
South or East Asian	9	4.0
Middle Eastern	1	0.4
Aboriginal	5	2.2
Latin American	3	1.3
Other or prefer not to answer	8	3.2
Employment Status of Parent Completing Survey		
Full-time	159	71.3
Part-time	26	11.7
Occasional/Support	3	1.3
Unemployed	28	12.6
Prefer not to answer	7	3.1
Family Situation		
Single parent	28	12.3
Double parent	191	86.0
Prefer not to answer	1	0.5
Other	2	1.2
Highest Level of Education of Parent Completing Survey		
High school	16	7.2
College	43	19.3
University	78	35.0
Graduate School	86	38.6
Housing Type		
Apartment	11	4.9
Condo	3	1.3
Townhouse	15	6.7
Semi-detached house	15	6.7
Detached house	177	79.4
Other	2	0.9
Presence of Family Dog		
Yes	88	41.9
No	122	58.1
Annual Household Income		
<$20,000	3	1.3
$20,000–$59,999	26	11.7
$60,000–$99,999	44	19.7
$100,000–$139,999	65	29.2
≥$140,000	71	31.8
Number of Children Under 12 Years of Age		
1	92	41.4
2	96	43.2
≥3	34	15.4

**Table 2:** Parents’ (*n =* 382) Perspectives of their Children’s Physical Activity Experiences and Behaviors.

	*n*	%
How often did your children ask about returning to organized sport/play in the neighborhood when these activities were either closed or deemed inaccessible by public health guidelines?		
*More than 3x per week*	170	44.5
*Seldom (1 − 2x) per week*	149	39.0
*Never*	63	16.5
Now that public health measures are being lifted in Ontario, are your children interested in the same activities/sports they engaged in prior to the pandemic, or different ones?		
*Same activities they were enrolled in prior to COVID*	154	40.4
*Same activities with the addition of new ones*	179	47.0
*Completely different activities*	27	7.1
*My children are no longer interested in returning to sport/other activities*	21	5.5
How often did you engage in physical activity with your children during closures of supportive spaces?		
*Never*	4	1.0
*Rarely*	27	7.1
*Sometimes*	80	20.9
*Often (weekly)*	152	39.8
*Very often (daily)*	119	31.2
If applicable, what helped you to support your children’s activity during the pandemic?		
*Personal interest in being active*	204	
*Having access to outdoor space(s)*	306	
*Virtual opportunities*	73	
*Other*	32	
*It was very difficult for me to support my children’s activity*	37	
Have you noticed any behavior changes in your children during the last 18 months?		
*Yes*	251	65.9
*No*	91	23.9
*Unsure*	39	10.2
Did you experience any challenges regarding supporting your children’s activity while at home?		
*Yes*	221	73.7
*No*	79	26.3
Did you and your children form any mini bubbles over the last 18 months?		
*Yes*	273	71.7
*No*	103	27.0
*Prefer not to answer*	5	1.3
Some activities were being conducted virtually during the pandemic. Are there any virtual activities/components that you would like to see continued?		
*Yes*	80	24.9
*No*	241	75.1
If you have returned your children to sport/activities, have you noticed any changes in your children’s physical activity levels?		
*Yes*	193	64.1
*No*	108	35.9
How concerned are you about your children’s physical activity levels following COVID-19?		
*Very concerned*	86	22.9
*Somewhat concerned*	136	36.3
*Not very concerned*	113	30.1
*Not at all concerned*	40	10.7

**Table 3:** Parents’ Open-Ended Responses Regarding their Children’s Movement Behaviors During the COVID-19 Pandemic in Ontario.

Question	Common Response	Supporting Quote
Please describe any differences (if any) in your children’s physical activity levels on weekdays compared to weekends	Lower levels of activity on weekdays due to work/school demands	- *“Too much homework during the week. More active on the weekends.”* - *“Spent so much time doing online learning during the week, sitting at computer. On weekends go for bike rides, hikes, etc.”* - *“Mon–Fri kids were busy with school and parents were busy working or supporting their at-home learning; evenings often reserved for a focus on work for parents. As such, weekends ended up being the best time for physical activity.”*
	More active during the week at childcare or school	- *“Weekdays I required her to spend a certain amount of active time as part of her school day.”* - *“Weekdays were at daycare which has an outdoor space.”* - *“During ’recesses’ of full-remote learning, we made a point of having children out-side playing.”* - *“When the schools were closed, we would make an effort to get out during the week (hikes, parks, pools) when it was not as busy as weekends.”*
	More sedentary time during the week because of screens	- *“Spent so much time doing online learning during the week, sitting at computer.”* - *“Screens on weekdays with school, limited screens on weekends.”* - *“They were addicted to screens Monday–Friday because my husband and I had to work. We tried to make up for it on the weekend with activities like cycling and hiking.”*
With respect to behavior changes (if any) during closures of supportive spaces, what did you notice in your children during the last 18 months?	Increased screen time	- *“Less motivated to be active more interest in screen time.”* - *“He wants to spend more time using digital screens, and it is more difficult to convince him to go out.”* - *“They spend way more time in front of screens and depending on what they watch, it affects their behavior.”*
	Lack of socializing and anxiety toward social situations	- *“My daughter became very anxious and was extremely worried about leaving the house, entering buildings, etc.”* - *“More anxiety about connecting with kids in the neighborhood once he was allowed to do so.”* - *“Anxiety about returning with peers.”* - *“My youngest is more withdrawn when we meet my friends, and she doesn’t engage.”*
	Increased general sadness/sense of loss	- *“Grumpier and a sense of loss.”* - *“My son felt very lonely, because he couldn’t meet with friends.”* - *“Sad, crying, frustration.”* - *“Sad not to have activities, missed friends, coaches.”*
	More easily agitated	- *“When he can’t run/move/shout/play outside he gets rangy (like stir crazy-naughty, disruptive behavior, jumping on furniture, climbing me, etc).”* - *“Youngest had more angry outbursts. More difficult to control.”* - *“Agitated behavior in older child when not given adequate opportunities to exercise, loss of desire to do activities or to get out of the house. Increased frustration.”*
	Separation anxiety from parents	- *“All the time with me has made him somewhat clingier.”* - *“Increased anxiety and social isolation leading to a clinginess to me.”* - *“My children, particularly my 9-year-old, are more needy and clingy.”*
What helped you to support your children’s activity during the pandemic?	Access to outdoor spaces	- *“Local community cleared ice on the lake to allow children to skate.”* - *“Having trees in my yard meant I could buy them swings and climbing apparatus to use at home.”* - *“We moved from a condo building with no outdoor space to a house with a large fenced in yards”*
	Support from friends and family	- *“Seeing friends in person, even if it was outside and the weather was terrible.”* - *“Low case #s in our region and a willingness of friends to participate in outdoor play.”*
	Access to sport/play-related resources	- *“Access to our own trampoline, sports equipment, and a private pool.”* - *“Money from government to buy a trampoline.”* - *“We bought a swing set and outdoor play equipment.”* - *“Having trees in my yard meant I could buy them swings and climbing apparatus to use at home.”*
Did you experience any challenges regarding supporting your children’s activity while at home? Please describe	Lack of space and/or equipment	- *“We live in an apartment with a small yard. We were lucky because our neighbors would let us play in their yard, but without this kindness, it would have been hard to find places to play on those cold winter days.”* - *“We don’t have a huge space, and what was enough for him at the start of the pandemic (3 years old) quickly became insufficient as he grew (now 5).”* - *“Space is limited. Can’t bat/throw a baseball inside the house. The yard is not big enough either.”* - *“Hard to get equipment.”*
	Children missing their peers	- *“They would prefer to play sports with their peers.”* - *“Trying to get them motivated to go outside when they couldn’t play with friends.”* - *“Periods where we couldn’t meet up with other children were difficult.”* - *“Sometimes harder to motivate to participate with just immediate household members. when wanted to be active with friends.”*
	Lack of time and work demands	- *“Working from home and supporting children is difficult.”* - *“Both myself and my spouse work full time, and during the first 6 months of the pandemic we had an infant at home full time as well. Physical activity had to work around those factors.”* - *“I’m working. Trying to keep them busy and do my job is very challenging”*
	Parental burnout/inability to co-participate	- *“I have a disability that doesn’t allow me to always keep up with him, as he has gotten older he wants someone to join in with him.”* - *“I myself am very out of shape and my feet hurt constantly. Was difficult to encourage them to play when I could not.”* - *“I personally do not exercise or workout, so I had to make a concerted effort to get out and be active with the kids.”* - *“I’m exhausted. I used to like to run and play with them, but now I’m too tired. It’s not fun anymore.”* - *“My own mental health was strained. Constant 24/7 responsibility for everyone’s health and happiness with no help or break was very difficult.”*
Due to COVID-19-associated closures, some activities (i.e., sports) were being conducted via a virtual space (e.g., Zoom, Skype, etc.). Now that a lot of these activities have begun opening back up, are there any components of these virtual activities that you would like to see continued?	Children and/or parents not interested in virtual opportunities	- *“I hope to never have any virtual activities ever again.”* - *“We are done with everything virtual.”* - *“My kids prefer in person activities and interacting with teammates and coaches.”* - *“My kids showed only minimal interest in virtual activities. The interest was not sustained.”*
	Virtual opportunities increase screen time	- *“They need to be off electronics!”* - *“Time away from screens is necessary. They need the social aspect not behind a screen.”*
	Virtual opportunities do not offer the same social, physical and/or developmental opportunities	- *“For my child, it was hard to connect with other children over Zoom”* - *“Online is ridiculous and doesn’t allow proper socialization or connection with other kids and parents.”* - *“Children need to be face to face and engaging with others in person and not over screens.”* - *“Prefer the social involvement and team/group aspect of being involved in in person physical activities.”*
	Virtual opportunities were convenient/safe	- *“It was nice being able to make supper while the class was going on and not have to drive there.”* - *“I think the virtual sessions remain a good option for kids who aren’t ready to head back for in person lessons but still want to do the classes with their friends.”* - *“The ability to engage in social and fun activity when weather is poor, or health conditions require such an approach would be appreciated long term.”* - *“Virtual if weather not cooperative.”* - *“Virtual activities are so much easier to accommodate into our schedules! Less driving around.”*
If you have returned your children to sport/activities, have you noticed any changes in your children’s physical activity levels?	Children not yet returned	- *“We are waiting for the vaccine to become available.”* - *“They have not returned—they have lost interest in soccer and baseball.”* - *“We have not returned to any in-person sports.”* - *“Hockey has not started, but knowing they get to play keeps them going.”*
	No changes since having returned	- *“They always feel great afterwards and enjoy being there.”* - *“Kids are kids. They are always full of energy!”*
	Changes in motivation to get active	- *“Yes, appears to have more energy and motivation.”* - *“They still resist getting active. They do enjoy doing activities with other kids, though.”* - *“Even higher interest and enjoyment in physical activity.”* - *“Trying harder and pushing themselves to do better.”*
	More active since having returned	- *“They are more intently active for longer periods.”* - *“They are more active now that organized sports have returned.”* - *“Hockey has not started, but knowing they get to play keeps them going.”*
	Decreased strength and endurance	- *“Their physical endurance and strength have diminished.”* - *“Not able to perform at same levels, tires easily … need to build back up.”* - *“They get tired more easily and out of breath.”* - *“Not in the same physical shape!”*
	Sleeping better since returned to play/sport	- *“Slowly returning to similar weight as he gained weight before, and also sleeps better now.”* - *“More tired at night after lessons.”* - *“Energy is being utilized in a positive way, she can sleep better at night and can focus on direction better.”*

## Data Availability

Not applicable.

## Supplementary Materials

The following supporting information can be downloaded at: https://www.mdpi.com/article/10.3390/ijerph192215061/s1, File S1: Items from the *Return to Play* study used for the present study, labelled by survey (e.g., baseline or follow-up survey).
