# Supplementary material for: Children’s Physical Activity during COVID-19 in Ontario, Canada: Parents’ Perspectives
Source: Int J Environ Res Public Health. 2022 Nov 16;19(22):15061. doi: 10.3390/ijerph192215061 (PMC9691234; doi:10.3390/ijerph192215061)
Supplement: Supplementary file 1 [file ijerph-19-15061-s001.zip › ijerph-2010576-supplementary.pdf]

Supplementary File S1. Items from the *Return to Play* study used for the present study, labelled by survey (e.g., baseline or follow-up survey).

Baseline Return to Play Post-COVID Survey Items

Q5.1 Please answer the following questions to provide some information about yourself.

Q5.2 What gender do you identify with? (Refers to **current gender** which may be different from sex assigned at birth and may be different from what is indicated on legal documents.)

- ○ Male (1)
- ○ Female (2)
- ○ Prefer not to say (3)
- ○ Prefer to self-describe: (4) ____

Q5.3 What is **your age** (in years)?

____

Q5.4 What is your postal code? (e.g., 1A1 A1A)?

____

Q5.5 Which of the following best describes the area you live?

- ○ Rural (1)
- ○ Suburban (2)
- ○ Urban (3)

Q5.6 What is your racial background/ethnicity?

- ◯ Caucasian (1)
- ◯ African Canadian (2)
- ◯ South Asian (3)
- ◯ East Asian (4)
- ◯ Middle Eastern (5)
- ◯ First Nations/Aboriginal (6)
- ◯ Latin American (7)
- ◯ Other: (8) ____
- ◯ Prefer not to answer (9)

Q5.7 What is your current employment status?

- ◯ Full-time (1)
- ◯ Part-time (2)
- ◯ Occasional/Support (3)
- ◯ Unemployed (4)
- ◯ Prefer not to answer (5)

Q5.8 What is your family situation?

- Single-parent (1)
- Double-parent (2)
- Guardian-led (3)
- Other: (4) ____
- Prefer not to answer (5)

Q5.9 What is your highest level of education?

- High school (1)
- College (2)
- University (3)
- Graduate school (4)
- Prefer not to answer (5)

Q5.10 In what housing type do you live (during the COVID-19 pandemic)?

- ◯ Apartment (1)
- ◯ Condominium (2)
- ◯ Townhouse (3)
- ◯ Semi-detached house (4)
- ◯ Detached house (5)
- ◯ Other housing? Please describe: (6) ____

Q5.11 Do you have a dog?

- ◯ Yes (1)
- ◯ No (2)

Q5.12 What is your approximate **yearly total household income (before taxes)**?

- Less than $20,000 (1)
- $20,000 - $39,000 (2)
- $40,000 - $ 59,000 (3)
- $60,000 - $79,000 (4)
- $80,000 - $99,000 (5)
- $100,000 - $119,000 (6)
- $120,000 - $139,000 (7)
- More than $140,000 (8)
- Prefer not to answer (9)

Q5.13 Ontario is re-opening public spaces in three stages. What phase of re-opening are you **currently** in?

- Phase 1 re-opening (1)
- Phase 2 re-opening (2)
- Phase 3 re-opening (3)

Q5.14 How many children aged 0-12 years do you currently provide care for?
Click to write Choice 1 (1)

▼ 1 (1) ... 10 (10)

Display This Question:

If How many children aged 0-12 years do you currently provide care for? = 1

Q5.15 What is the current age (0-12 years) and sex at birth of your child?

	Male (1)	Female (2)
Age of child: (1)	☐	☐

Display This Question:

If How many children aged 0-12 years do you currently provide care for? = 2

Q5.16 What is the current age (0-12 years) and sex at birth of your children? Please complete the fields below in order from **youngest to oldest.**

	Male (1)	Female (2)
Child 1. Age of child: (1)	☐	☐
Child 2. Age of child: (2)	☐	☐

Display This Question:

If How many children aged 0-12 years do you currently provide care for? = 3

Q5.17 What is the current age (0-12 years) and sex at birth of your children? Please complete fields below in order from youngest to oldest.

	Male (1)	Female (2)
Child 1. Age of child: (3)	☐	☐
Child 2. Age of child: (4)	☐	☐
Child 3. Age of child: (5)	☐	☐

Display This Question:

If How many children aged 0-12 years do you currently provide care for? = 4

Q5.18 What is the current age (0-12 years) and sex at birth of your children? Please complete fields below in order from youngest to oldest.

	Male (1)	Female (2)
Child 1. Age of child: (1)	☐	☐
Child 2. Age of child: (2)	☐	☐
Child 3. Age of child: (3)	☐	☐
Child 4. Age of child: (4)	☐	☐

Display This Question:

If How many children aged 0-12 years do you currently provide care for? = 5

Q5.19 What is the current age (0-12 years) and sex at birth of your children? Please complete fields below in order from youngest to oldest.

	Male (1)	Female (2)
Child 1. Age of child: (1)	☐	☐
Child 2. Age of child: (2)	☐	☐
Child 3. Age of child: (3)	☐	☐
Child 4. Age of child: (4)	☐	☐
Child 5. Age of child: (5)	☐	☐

Display This Question:

If How many children aged 0-12 years do you currently provide care for? = 6

Q5.20 What is the current age (0-12 years) and sex at birth of your children? Please complete fields below in order from youngest to oldest.

	Male (1)	Female (2)
Child 1. Age of child: (1)	▢	▢
Child 2. Age of child: (2)	▢	▢
Child 3. Age of child: (3)	▢	▢
Child 4. Age of child: (4)	▢	▢
Child 5. Age of child: (5)	▢	▢
Child 6. Age of child: (6)	▢	▢

Display This Question:

If How many children aged 0-12 years do you currently provide care for? = 7

Q5.21 What is the current age (0-12 years) and sex at birth of your children? Please complete fields below in order from youngest to oldest.

	Male (1)	Female (2)
Child 1. Age of child: (1)	☐	☐
Child 2. Age of child: (2)	☐	☐
Child 3. Age of child: (3)	☐	☐
Child 4. Age of child: (4)	☐	☐
Child 5. Age of child: (5)	☐	☐
Child 6. Age of child: (6)	☐	☐
Child 7. Age of child: (7)	☐	☐

Display This Question:

If How many children aged 0-12 years do you currently provide care for? = 8

Western UNIVERSITY · CANADA

Child Health & PHYSICAL ACTIVITY LABORATORY

Q5.22 What is the current age (0-12 years) and sex at birth of your children? Please complete fields below in order from youngest to oldest.

	Male (1)	Female (2)
Child 1. Age of child: (1)	☐	☐
Child 2. Age of child: (2)	☐	☐
Child 3. Age of child: (3)	☐	☐
Child 4. Age of child: (4)	☐	☐
Child 5. Age of child: (5)	☐	☐
Child 6. Age of child: (6)	☐	☐
Child 7. Age of child: (7)	☐	☐
Child 8. Age of child: (8)	☐	☐

Display This Question:

If How many children aged 0-12 years do you currently provide care for? = 9

Western UNIVERSITY · CANADA

Child Health & PHYSICAL ACTIVITY LABORATORY

Q5.23 What is the current age (0-12 years) and sex at birth of your children? Please complete fields below in order from youngest to oldest.

	Male (1)	Female (2)
Child 1. Age of child: (1)	☐	☐
Child 2. Age of child: (2)	☐	☐
Child 3. Age of child: (3)	☐	☐
Child 4. Age of child: (4)	☐	☐
Child 5. Age of child: (5)	☐	☐
Child 6. Age of child: (6)	☐	☐
Child 7. Age of child: (7)	☐	☐
Child 8. Age of child: (8)	☐	☐
Child 9. Age of child: (9)	☐	☐

Display This Question:

If How many children aged 0-12 years do you currently provide care for? = 10

Q5.24 What is the current age (0-12 years) and sex at birth of your children? Please complete fields below in order from youngest to oldest.

	Male (1)	Female (2)
Child 1. Age of child: (1)	☐	☐
Child 2. Age of child: (2)	☐	☐
Child 3. Age of child: (3)	☐	☐
Child 4. Age of child: (4)	☐	☐
Child 5. Age of child: (5)	☐	☐
Child 6. Age of child: (6)	☐	☐
Child 7. Age of child: (7)	☐	☐
Child 8. Age of child: (8)	☐	☐
Child 9. Age of child: (9)	☐	☐
Child 10. Age of child: (10)	☐	☐

Q9.1 **How often** did your children ask about returning to organized sport and/or play in the neighbourhood when these activities were either closed or deemed inaccessible by public health guidelines?

- ◯ More than 3x per week (1)
- ◯ Seldom (1-2x per week) (2)
- ◯ Never (3)

Q9.2 Now that public health measures are being lifted in Ontario, **are your children interested in the same activities and/or sports** they engaged in prior to the pandemic, **or different ones?**

- ◯ Same activities they were enrolled in prior to COVID-19 (1)
- ◯ Same activities with the addition of new ones (2)
- ◯ Completely different activities (3)
- ◯ My child(ren) are no longer interested in returning to sport/activities (4)

Q9.3 **How often did you engage in physical activity with your child(ren) during closures of supportive spaces (i.e., community centres, sport fields, parks, etc.)?**

- ◯ Never (1)
- ◯ Rarely (2)
- ◯ Sometimes (3)
- ◯ Often (weekly) (4)
- ◯ Very often (daily) (5)

Q9.4 **In your opinion**, were your children more active during **weekdays** (i.e., Monday-Friday) or **weekends** (i.e., Saturday-Sunday) **during the last 18 months (i.e., March 2020- Present day)?**

- ○ Weekdays (1)
- ○ Weekends (2)

Q9.5 **Please describe** any differences (if any) in your child(ren)'s physical activity levels on weekdays (i.e., Monday-Friday) compared to Weekends (Saturday-Sunday)

____

Q9.6 **Have you noticed any behaviour changes in your child(ren) during the last 18 months?**

- ○ Yes (4)
- ○ No (5)
- ○ Unsure (6)

Q9.7 With respect **behaviour changes (if any) during** closures of supportive spaces (i.e., community centres, sports fields, parks, etc.), **what did you notice in your child(ren) during the last 18 months?**

____

Q9.8 If applicable, **what helped you to support your child(ren)'s activity during the pandemic?**

- ☐ Personal interest in being physically active (1)
- ☐ Access to outdoor spaces (2)
- ☐ Virtual opportunities (i.e., virtual sport lessons, YouTube, etc.) (3)
- ☐ Other. Please describe: (4) ____
- ☐ It was very difficult for me to support my child(ren)'s physical activity (5)

Q9.9 Did you experience **any challenges** with regard to supporting your child(ren)'s activity while at home? **Please describe.**

- ○ Yes. Please describe: (4) ____
- ○ No. Please describe: (5) ____

Q9.10 Did **you and your child(ren)** form any mini bubbles (i.e., have designated people and/or families that you would interact with) **over the last 18 months (i.e., March 2020 - present day)?**

- ○ Yes (1)
- ○ No (2)
- ○ Prefer not to answer (3)

Q9.11 Due to COVID-19 associated closures, some activities (i.e., sports) were being conducted via a **virtual space** (e.g., Zoom, Skype, etc.). Now that a lot of these activities have begun

opening back up, **are there any components of these virtual activities that you would like to see continued?**

○ Yes. Please describe: (4) ____

○ No. Please describe: (5) ____

Q9.12 If you have returned your child(ren) to sport/activities, **have you noticed any changes in your child(ren)'s physical activity levels?**

○ Yes. Please describe: (1) ____

○ No. Please describe: (2) ____

Q9.13 How concerned are you about your child(ren)’s physical activity levels following COVID-related closures/ lockdowns?

○ Very concerned (1)

○ Somewhat concerned (2)

○ Not very concerned (4)

○ Not at all concerned (5)

End of Block: Block 5
